# Supplementary material for: Comparative multiomics analysis of cell physiological state after culture in a basket bioreactor
Source: Sci Rep. 2022 Nov 23;12:20161. doi: 10.1038/s41598-022-24687-4 (PMC9686226; doi:10.1038/s41598-022-24687-4)

23666045.1:75124019:75124094:-@NW_023666045.1:75124019:75125038:-@NW_023666045.1:75127235:7512

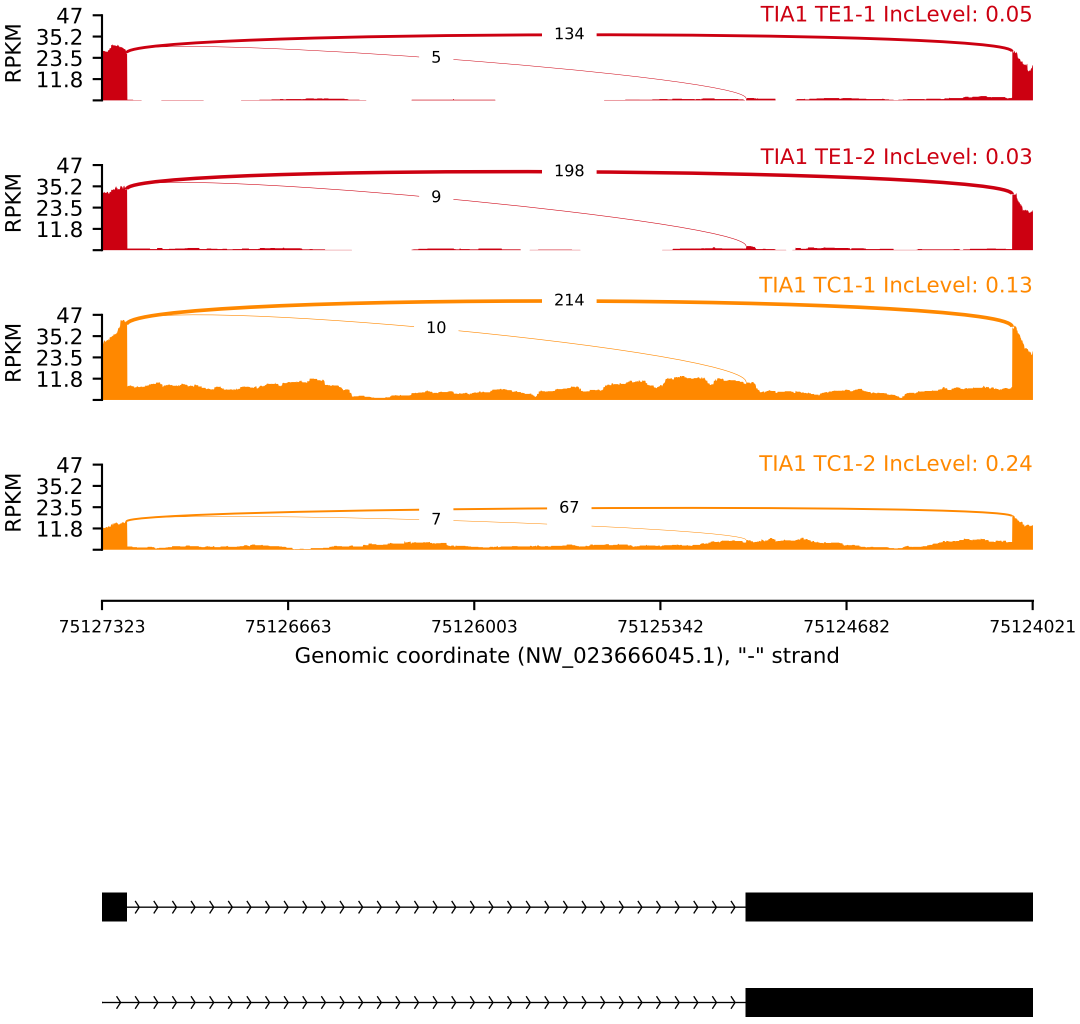

Supplement: Supplementary file 1 — Supplementary Information 1. [file 41598_2022_24687_MOESM1_ESM.zip › raw data/Transcriptomic raw data/7.AS/2.dasplot/TE1vsTC1/A3SS/18_TIA1_NW_023666045.1_75124019_75124094_-@NW_023666045.1_75124019_75125038_-@NW_023666045.1_75127235_75127322_-.pdf]
